# Gemcitabine in combination with epibrassinolide enhanced the apoptotic response in an ER stress-dependent manner and reduced the epithelial-mesenchymal transition in pancreatic cancer cells

**DOI:** 10.55730/1300-0152.2630

**Published:** 2022-09-19

**Authors:** Pınar OBAKAN YERLİKAYA, Leila MEHDIZADEHTAPEH, Özge RENCÜZOĞULLARI, Fadina KURYAYEVA, Sena Sedef ÇEVİKLİ, Şevval ÖZAĞAR, Sibel Pınar ODABAŞ, Sude TUNÇKOL, Hakan YETİM, Ajda ÇOKER GÜRKAN, Elif Damla ARISAN

**Affiliations:** 1 Department of Molecular Biology and Genetics, Faculty of Engineering and Natural Sciences, İstanbul Medeniyet University, İstanbul, Turkey; 2 Science and Advanced Technology Research Center, İstanbul Medeniyet University, İstanbul, Turkey; 3 Department of Food and Nutrition, İstanbul Sabahattin Zaim University, İstanbul, Turkey; 4 Department of Molecular Biology Genetics, İstanbul Kültür University, İstanbul, Turkey; 5 Department of Biology, Faculty of Science and Literature, Marmara University, İstanbul, Turkey; 6 Institute of Biotechnology, Gebze Technical University, Kocaeli, Turkey

**Keywords:** Pancreatic cancer, endoplasmic reticulum stress, epibrassinolide, gemcitabine

## Abstract

Gemcitabine is a broad-spectrum antimetabolite and a deoxycytidine analog recognized as a standard therapy alone or in combination with other antineoplastic agents in the therapy of pancreas cancer. Drug resistance following gemcitabine treatment is a common phenomenon; therefore, combinational therapy models are usually preferred. Pancreatic ductal adenocarcinoma, or pancreas cancer, is the fourth leading cause of cancer-related deaths worldwide. With the increasing incidence of pancreatic cancer every year, the mortality rate is also rising significantly because of late diagnosis, and limited chemotherapy options. Adjuvant chemotherapy after surgical resection is the typical option for the treatment of early pancreatic cancer. Mostly, 5-fluorouracil/leucovorin with irinotecan and oxaliplatin (FOLFIRINOX) and gemcitabine/nab-paclitaxel is used for the prognosis of advanced pancreatic cancer; however, chemoresistance usually occurs limiting the effectiveness of the chemotherapy. Therefore, most of the studies are focused on gemcitabine combination with other drugs to overcome the situation.

As an apoptotic agent and a member of brassinosteroids, epibrassinolide (EBR) induces endoplasmic reticulum (ER) stress-dependent cell death in different cancer cells, as shown by our group. In this study, we aimed to enhance the gemcitabine apoptotic effect by EBR combined treatment in pancreatic cancer cells. EBR treatment reduced cell viability and inhibited cell proliferation in PANC-1, MIA PaCa-2, and AsPC-1 cells. Each pancreatic cancer cell gave different responses to the EBR treatment because of different aggressiveness. However, EBR induced apoptosis through increasing ROS generation, which was associated with ER stress in PANC-1 and MIA PaCa-2 cells. Gemcitabine alone reduced the cell viability of each pancreatic cancer cell line; however, combination with EBR led to further induction of apoptotic cell death in each pancreatic cancer cell line. In addition, combined treatment of gemcitabine and EBR further decreased N-cadherin and vimentin expressions, suggesting that epithelial-mesenchymal transition of pancreatic cells is reduced. In conclusion, EBR had therapeutic potential to avoid the gemcitabine-induced side effects during the treatment of pancreatic cancer.

## 1. Introduction

The pancreas, as a secretory nodular gland, is a critical center of energy consumption and metabolism of the gastrointestinal system. Pancreatic cancer, with poor outcomes, keeps one of the most malignant neoplasms as well as being the most lethal condition, particularly in developed countries. The two major types of pancreatic cancer are ductal adenocarcinoma (PDAC) which includes about 85% of cases, and less than 5% of all cases are pancreatic neuroendocrine tumors (NETs) (McGuigan et al., 2018). Early detection is considered the most efficient way to boost survival in pancreatic cancer. Unfortunately, tumor formation and development are asymptomatic in general, and the disease only becomes conspicuous after metastasis (Melisi et al., 2018). Pancreatic cancer has been separated into subtypes according to the transcription factors profiles, integrated genomic analysis, the prevalence of aberrations in key driver genes, core signaling pathways, and downstream targets. The next-generation sequencing data from PDAC patients showed mostly mutated genes playing a role in cell cycle progression, DNA repair, RNA processing, chromatin modification, tumor suppressors, etc. Especially K-Ras, TGF-β, Wnt, Notch, and SWI-SNF signaling pathways have been shown to be altered during PDAC (Pelosi et al., 2017). Resistance mechanisms improved by tumors and unfavorable responses to current therapies emphasize the need to evolve alternative treatment approaches. Gemcitabine is an anticancer nucleoside and the analog of deoxycytidine. Once gemcitabine is transported to the cell, it is phosphorylated by deoxycytidine kinase to an active form. It exerts the antitumor effect by suppressing the activity of ribonucleotide reductase, thus inhibiting DNA synthesis and blocking cell proliferation and division (Plunkett et al., 1995). The clinical benefit of gemcitabine is approximately five times higher than 5-fluorouracil (5-FU): although its effect on survival is limited. The poor penetration of the drug into the hyper vascularized, dense tumor stroma and the development of chemoresistance restrict the use of gemcitabine and it is usually preferred in combinational therapy models (Oettle & Riess, 2002). It has been shown that Akt, epithelial growth factor receptor (EGFR): MAPK signaling pathways involved in the formation of gemcitabine resistance, and cells in the epithelial-mesenchymal transition (EMT) phenotype play an active role in pancreatic cancer chemoresistance. EMT is a cellular process during which cells change their morphology to lose epithelial, and gain mesenchymal properties and metastatic profile via enhanced migratory capacity, invasiveness, and elevated resistance to apoptosis. EMT is characterized by the downregulation of E-cadherin, a parallel increase in the expressions of N-cadherin and vimentin (Sommariva & Gagliano, 2020). These protein expression alterations are provided by transcription factors Slug, Snail, Zeb1/2, and SMAD-interacting protein 1. Different mechanisms like cytokine signaling, growth factors, and endoplasmic reticulum stress can lead to EMT in different carcinomas, including PDAC (De Wever et al., 2008).

In eukaryotic cells, the biosynthesis, folding, and modifications of secretory and membrane proteins are conducted by the endoplasmic reticulum (ER) (Benham, 2019). Cellular redox regulation disturbance, glucose deprivation, viral infections, and chemotherapeutics can cause an accumulation of un-/misfolded proteins in the ER, triggering an evolutionarily conserved response termed the unfolded protein response (UPR). To maintain cell homeostasis under stress conditions, UPR induces certain signaling pathways to support protein folding, decrease translation activity to limit unprocessed protein amounts, and degrade un-/misfolded protein through the ubiquitin-proteasome pathway. These decisions are made following the chaperone protein GRP78/BiP dissociation from three transmembrane sensor proteins in the ER, namely PERK, IRE1α, and ATF6 (Wang & Kaufman, 2012). The PERK-mediated response includes ATF4 activation following eIF2α phosphorylation, resulting in CHOP translocation to the nucleus. CHOP is a critical player in the decision of apoptotic cell death by repressing the antiapoptotic protein Bcl-2 and activation of proapoptotic BH3 domain-only proteins such as Bax. The IRE1-mediated ER stress response initiates with oligomerization and autophosphorylation following Grp78/BiP dissociation. Once IRE1α is autophosphorylated, its endoribonuclease activity is induced and splices XBP-1 mRNA to produce an active XBP-1 protein. The mature protein is a transcription factor for genes involved in protein homeostasis. IRE1α signaling can also stimulate Jun-N-Terminal kinase (JNK) and p38 via induction of apoptotic signaling kinase 1 (ASK1). JNK and p38 have been shown to promote the phosphorylation and activation of proapoptotic Bax. The third ER stress-induced signaling cascade begins with ATF6 release from Grp78/BiP and translocation to the Golgi followed by proteolytic cleavage of the N-terminal fragment processed by Site-1 (S1P) and Site-2 (S2P) proteases. Upon maturation, ATF6 is transported into the nucleus to turn on gene expression such as CHOP, GRP78, and XBP1. ATF6 signaling also triggers JNK, Rheb/mTOR, and apoptosis pathways. Besides, it has been demonstrated that caspase-12, which is directly activated by ER stress, is translocated from the ER to the cytoplasm and causes the activation of caspase-9, known as the cell death initiating caspase (Lin et al., 2019).

Brassinosteroids (BR) are a group of steroids involved in plant growth, development, and stress responses (Nolan et al., 2020). It is noteworthy that BRs have also remarkable biological activity such as antiangiogenic, anticancer, antigenotoxic, antiviral, antifungal, and antibacterial in animal systems shown in both in vitro and in vivo studies (Kohli et al., 2020). Our recent studies showed that epibrassinolide (EBR): a member of BRs exerts an apoptotic effect in cancer cells by modulating several signaling pathways and metabolisms including PI3K/Akt and polyamines. Our recent studies enlighten the main target of EBR as calreticulin (CALR): an endoplasmic reticulum resident protein playing a role in protein folding. The SILAC (stable isotope labeling with amino acids in cell culture) assay and verification experiments via overexpression and silencing CALR have indicated that UPR and ER stress play a crucial role in EBR-induced apoptosis [13][14]. Besides, due to the natural origin of EBR, it does not induce toxic effects in mouse embryonic fibroblasts (MEF) or human fetal colon epithelial cells at micromolar concentrations, unlike cancer cells. Our aim in this study is to check the ability of EBR to enhance gemcitabine sensitivity in pancreatic cells by investigating apoptotic signaling pathways as well as the expressions of drug resistance influencing ER stress and EMT process proteins.

## 2. Materials and methods

### 2.1. Materials

EBR was purchased from Apollo Scientific (Manchester, UK) and dissolved in DMSO (stock concentration 5 mM). Gemcitabine was purchased from Sigma Aldrich (St. Louis, MO, USA) and prepared at a 150 mM stock concentration. Caspase 9, Caspase 7, Bcl-2, Bax, Puma, Bim, Bak, Bid, BiP, CALR, CALNX, PDI, IRE1a, PERK, Ero1La antirabbit or antimouse antibodies were purchased from Cell Signaling Technology (each 1:1000, CST, Danvers, MA, USA). Loading control B-actin and GAPDH antibodies and HRP-conjugated secondary antirabbit and antimouse were purchased from CST.

### 2.2. Cell lines and cell culture

MIA PaCa-2, PANC-1, and AsPC1 cells were purchased from American Tissue and Cell Culture (ATCC). MIA PaCa-2 and PANC-1 cells were grown in DMEM medium and AsPC1 cells in RPMI medium with the addition of 10% fetal bovine serum and 10 U/mL penicillin/streptomycin at 37° C in a humidified 5% CO2 incubator (HERAcell 150; Thermo Electron Corporation, Waltham, MA, USA).

### 2.3. Cell viability assay

Cells were seeded at a 1 × 10^4^ density of cells/well in 96-well plates and exposed to EBR and (or) GEM at different time points. Ten microliters of 3-(4,5-dimethylthiazol-2-yl)-2,5-diphenyl tetrazolium bromide dye (5 mg/mL) (Sigma-Aldrich) were added to each well and cells were kept at 37 °C for 4 h. The resulting formazan crystals were solubilized in 200 μL of dimethyl sulfoxide (DMSO). The density of the solubilized formazan was read at 570 nm spectrophotometrically (Bio-Rad, Hercules, CA, USA).

### 2.4. Colony formation assay

Cells were seeded at a 1 × 10^4^ density of cells/well into 6-well plates and treated with EBR and/or GEM. The media were then removed and cells were washed with 1X PBS fixed with methanol: acetic acid (3:1) for 5 min. Following the removal of fixing agents, cells were stained with 0.5% crystal violet in methanol for 15 min, washed with distilled water, and the morphological images were taken under light microscopy.

### 2.5. Trypan blue dye exclusion assay

Cells were seeded at a 1 × 10^5^ density of cells/well in 6-well plates (TPP, Zollstrasse, Switzerland) and treated with EBR and/or GEM time-dependently within 96 h. After trypsinization (Trypsin EDTA (0.25%): Gibco, USA): and centrifugation, cells were exposed to 0.4% (w/v) Trypan Blue (Gibco, USA) and cell culture media at a 1:1 ratio. Ten microliters of cells were counted by a dual-chamber 0.1 mm deep Neubauer improved hemocytometer.

### 2.6. Wound healing assay

Cells were seeded at a 1×10^6^ density of cells/well and grown to 80% confluence in 35 mm plates. The cell monolayer was then scratched with the narrow end of a sterile 200-μL pipette tip. Subsequently, the medium was promptly replaced to eliminate floating cells. The width of the scratch was measured at two points in each well after initial wounding. The cells were incubated for 24 h at 37 °C in a CO_2_ incubator, and then, the scratch width was remeasured. The relative motility and migration ability of the cells into the cell-free zone is expressed as the normalized percent change in the scratch width after 24, 48, and 72 h.

### 2.7. Immunoblotting

Cells were cultured in 60-mm Petri dishes in a complete medium. The media were then discarded and the cells were washed with ice-cold 1X PBS and lysed with ProteoJET Mammalian cell lysis buffer (Fermentas, St. Leon-Rot, Germany). For cytoplasmic and nuclear protein extraction NE-PER nuclear and cytoplasmic extraction kit (Thermo Scientific, Rockford, IL, USA) was used according to the manufacturer’s instructions. Total, cytoplasmic, and nuclear protein levels were determined by the Bradford method (Bio-Rad, Hercules, CA, USA). Total cell lysates were separated by 12% SDS–PAGE gels and transferred onto polyvinylidene difluoride (PVDF) membranes (Roche) subjected to electrophoresis. Membranes were washed in tris-buffered saline with Tween-20 (TBS-T) (10 mM Tris-HCl (pH 8.3): 0.05% Tween-20) (Tween 20, Sigma Ultra, St. Louis, MO, USA). Blocking was preceded by 5% skim milk containing TBS-T milk overnight at 4 °C. PVDF membranes were incubated with a primary antibody buffer containing 5% (v:v) skim milk solution with appropriate antibodies. Membranes were rinsed with TBS plus 0.05% v/v Tween-20 and incubated overnight with horseradish peroxidase (HRP)-conjugated secondary antibodies (antirabbit IgG, 1:5000 (v:v)) at 4 °C. Following the addition of an enhanced chemiluminescence reagent, signals from the HRP-coupled antibodies were detected using ChemiDoc MP Imaging System (Bio-Rad Laboratories, Hercules, CA). All results were replicated at least three times and representative blots were given.

### 2.8. Statistical analysis

All the experiments were statistically analyzed by GraphPad Prism 6 software (http://www.graphpad.com/). Error bars in the graphs were generated using ± standard deviation (SD) values. A statistical significance test was utilized by using ANOVA Bonferroni’s multiple comparisons test. p < 0.05 was taken as a level of significance. The results were repeated at least three times. The immunoblotting results shown are representative of three separate experiments.

## 3. Results

### 3.1. Epibrassinolide (EBR) treatment decreased cell viability, growth, and colony formation of pancreatic cells

**Figure 1:**
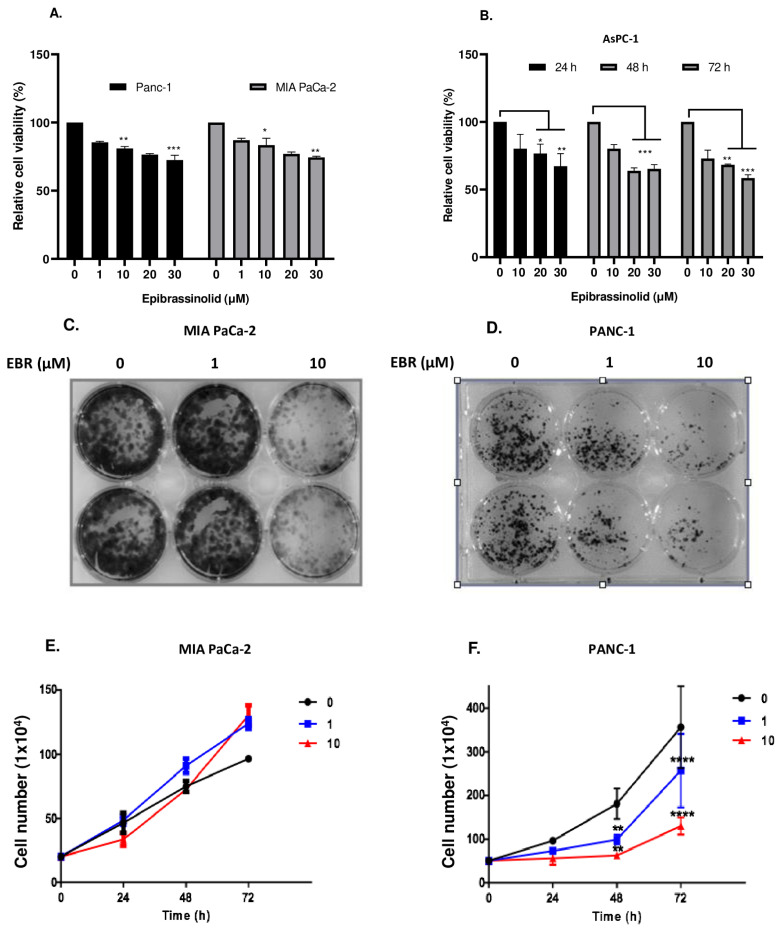
EBR treatment reduced the cell viability of pancreatic cancer cells. **A**. PANC-1 and MIA PaCa-2 cells were treated with EBR (1, 10, 20, 30 μM) for 24 h **B**. AsPC-1 cells were treated with 10, 20, 30 μM EBR for 24, 48, and 72 h. Afterward, the MTT cell viability assay was performed for each pancreatic cancer cell **p < 0.02, ***p < 0.001. **C**. 5 × 10^3^ cells/well were seeded into 6 well plates and treated with 1 and 10 μM EBR for 24 h. Subsequently, MIA PaCa-2 **D**. and PANC-1 cells were fixed and stained with crystal violet. Trypan blue dye exclusion assay was performed in **E**. MIA PaCa-2 and **F**. PANC-1 cells following 1 and 10 μM EBR treatments for 24, 48, and 72 h.

**Figure 2:**
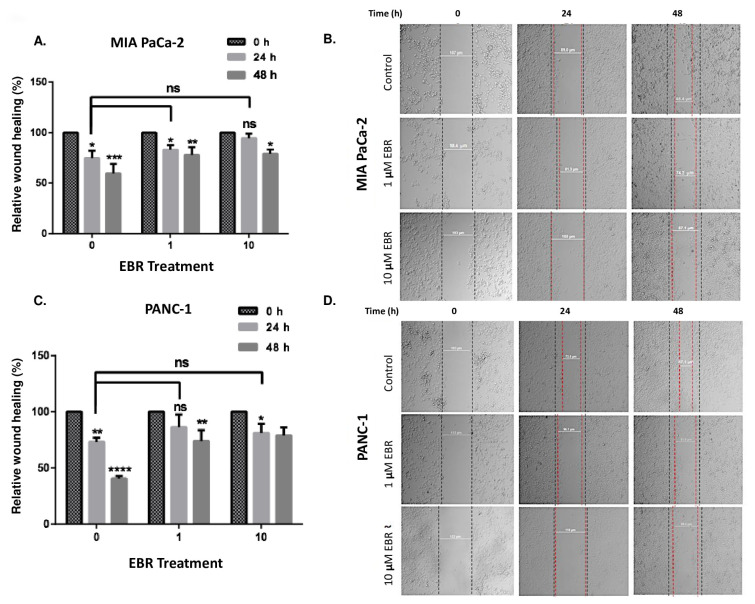
EBR treatment inhibited the cell migration capacity of pancreatic cancer cells. 5 × 10^5^ cells/well were seeded to 6 well plates and incubated until they reached 80% confluence. Wound Healing assay was performed following 1 and 10 μM EBR treatments of MIA PaCa-2 (A–B) and PANC-1 (C–D) cells for 24 h. The wound closure was calculated by Olympus IX70 light microscopy *p < 0.05, **p < 0.02, ***p < 0.001.

To evaluate the effect of EBR on the cell viability of pancreatic cells, MIA PaCa-2 and PANC-1 cells were treated with EBR in a dose-dependent manner (1–30 μM) for 24 h (Figure 1A). According to MTT cell viability assay results, while all concentrations of EBR treatment reduced the cell viability of MIA PaCa-2 cells significantly, a significant decrease in cell viability from 10 μM EBR concentration was observed in PANC-1 cells for 24 h. It was recorded that 1 and 10 μM EBR decreased cell viability by 20% and 40% in MIA PaCa-2 cells, respectively. While 10 μM EBR led to 45% of cell viability loss, there was no difference in 1 μM-treated PANC-1 cells. To understand the effect of EBR on more aggressive pancreatic cancer cells, AsPC-1 cells were cultured and treated with 10, 20, and 30 μM EBR for 24, 48, and 72 h (Figure 1B). The cell viability loss was recorded by 20% and 40% decrease after 24 h treatment of 10 and 20–30 μM EBR. The doubling time of AsPC-1 was around 38 h and the proliferation rate was higher than MIA PaCa-2 and PANC-1 cells. Therefore, 20 and 30 μM treatment of EBR decreased the cell viability by 30% and 40% for 48 h, while 40% and 45% for 72 h, respectively. To confirm these data, a trypan blue dye exclusion assay was performed. As shown in Figures 1C and 1D, the cytostatic effect of EBR treatment was observed in each cell line. While the proliferation ability of PANC-1 cells was strictly inhibited by EBR treatment, the proliferation rate of MIA PaCa-2 cells was slowed down until 48 h treatment. However, the effect of EBR for 72 h treatment was not effective for the inhibition of cell survival in MIA PaCa-2 cells. The long-term effect of EBR on MIA PaCa-2 and PANC-1 cells was investigated by colony formation assay (Figures 1E and 1F). MIA PaCa-2 and PANC-1 cells were treated with 1–10 μM EBR and it was observed that 1 μM EBR was enough to inhibit the colony-forming potential of the PANC-1 cells, but not in MIA PaCa-2 cells. As shown in Figures 1E and 1F, 10 μM EBR blocked the colony-forming ability of both MIA PaCa-2 and PANC-1 cells. Moreover, the effect of EBR on the migration potential of cells was investigated by wound healing assay. One and 10 μM EBR-treated and -untreated PANC-1 and MIA PaCa-2 cells were observed for 24 and 48 h. The distance in the wound area in untreated cells decreased significantly, whereas the closing rate of the wounds decreased by EBR treatment in both MIA PaCa-2 (Figures 2A and 2B) and PANC-1 cells (Figures 2C and 2D).

### 3.2. EBR induced apoptotic cell death in PANC-1 and MIA PaCa-2 cells

**Figure 3:**
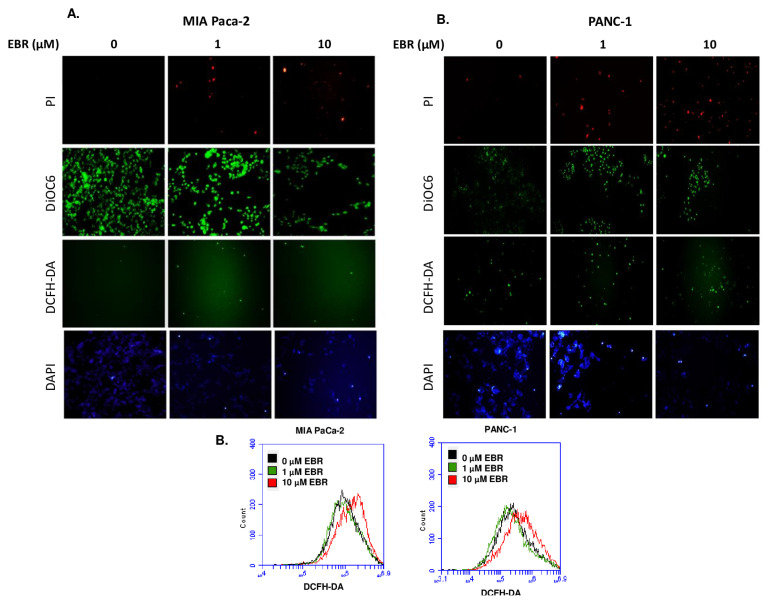

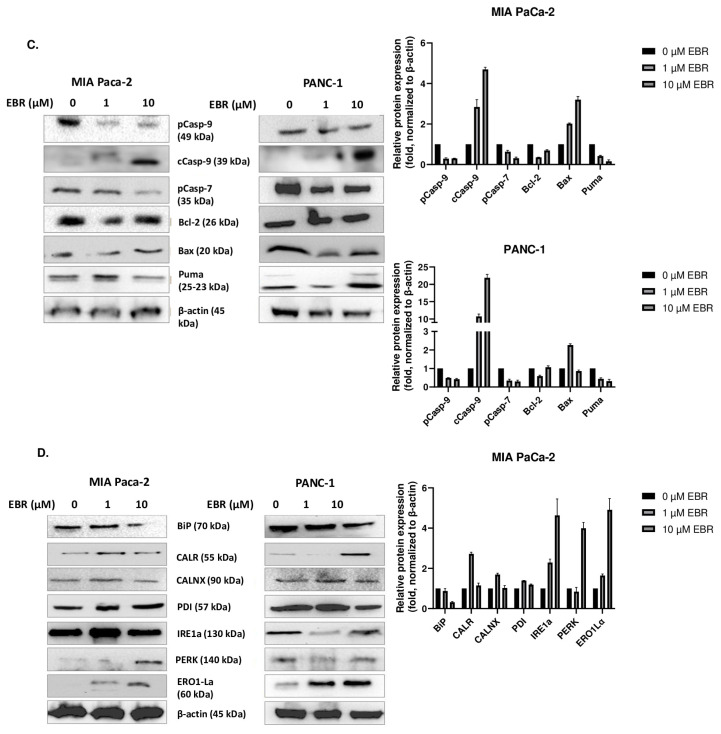
EBR triggered ER stress-induced apoptosis in a caspase-dependent manner in MIA PaCa-2 and PANC-1 cells. A. MIA PaCa-2 and PANC-1 cells were treated with 1 and 10 μM EBR for 24 h. Next, PI, DiOC6, DCFH-DA, and DAPI fluorescence staining proceeded. Stained cells were visualized by Olympus IX70 fluorescence microscopy **B**. Flow cytometric analysis of DCFH-DA staining (5 μM) was performed following EBR treatment of MIA PaCa-2 and PANC-1 cells. **C**. The expression levels of full caspase 9, cleaved caspase 9, full caspase 7, Bcl-2, Bax, and PUMA were determined by western blotting following total protein isolation. **D**. ER stress-related markers BIP, CALR, CALNX, PDI, IRE1α, PERK, and ERO1α were analyzed by western blotting in MIA PaCa-2 and PANC-1 cells following 1 and 10 μM EBR treatments. β-actin was used as a loading control.

To further investigate the mechanistic effect of EBR-induced cell death, EBR-treated cells were stained with PI, DiOC6, DCFH-DA, and DAPI (Figure 3A). The result of PI staining confirmed the MTT cell viability assay, in which increasing red dots showed death cells following 10 μM EBR treatment. EBR induced the mitochondrial membrane potential loss that led to a decrease in DiOC6 staining. The ROS generation was determined by fluorescence microscopy and flow cytometry following DCFH-DA staining (Figure 3B). While 1 μM EBR treatment of PANC-1 and MIA PaCa-2 cells resulted in no significant increase in ROS generation, 10 μM EBR treatment induced a prominent increase in ROS generation. Additionally, these results have attracted interest to determine the apoptotic role of EBR in pancreatic cancer cells. Therefore, the expression role of pro- and antiapoptotic markers was determined by western blotting (Figure 3C). PANC-1 and MIA PaCa-2 cells were treated with 1 and 10 μM EBR and total protein isolation was performed following 24-h treatment. It was observed that while procaspase 9 expression levels were significantly downregulated, the cleaved caspase-9 levels showed a remarkable increase after 10 μM EBR treatment in both MIA PaCa-2 and PANC-1 cells. Furthermore, 1 μM EBR treatment also resulted in a slight increase of cleaved caspase 9 in each cell line. The expression level of procaspase 7 was decreased in a dose-dependent manner in MIA PaCa-2 and PANC-1 cells (Figure 3C). Both Bax and Puma were proapoptotic members, while 10 μM EBR treatment increased the Puma levels in PANC-1 cells, there was an opposite effect in MIA PaCa-2 cells. However, Bax levels were decreased in EBR-treated PANC-1 and MIA PaCa-2 cells. EBR treatment did not exert any effect on the antiapoptotic Bcl-2 expression levels in each cell line.

### 3.3. EBR induced ER-stress related markers in PANC-1 and MIA PaCa-2 cells

EBR’s role to induce ER stress in pancreatic cancer cells was further evaluated. The expression levels of BiP, Calreticulin (CALR): Calnexin (CALNX): PDI, IRE1α, PERK, and Ero1α were analyzed by western blotting (Figure 3D). The ER stress is regulated by PERK and IRE-1α which are transmembrane receptors and their interaction with BIP makes these receptors inactive. Firstly, 10 μM EBR treatment increased the PERK levels, there was no difference in the Ire1a levels in PANC-1 and MIA PaCa-2 cells. However, 1 μM EBR treatment led to a significant decrease in IRE1α in PANC-1 cells and an increase in MIA PaCa-2 cells. Moreover, while 1 μM EBR did not alter the level of BiP, 10 μM EBR decreased its expression in both MIA PaCa-2 and PANC-1 cells (Figure 3D). CALR expression increased after 1 μM EBR significantly, but not following 10 μM EBR treatment in MIA PaCa-2 cells. The expression profile of CALR, on the other hand, increased significantly in a dose-dependent manner in PANC-1 cells. PDI can act with BiP to control misfolded protein levels in the cell. Compared to 10 μM EBR, 1 μM EBR treatment increased the PDI expression levels in MIA PaCa-2 cells more significantly. Contrarily, the expression of PDI was downregulated in PANC-1 cells. The Ero1α expression levels were increased following 1 and 10 μM EBR treatments of MIA PaCa-2 and PANC-1 cells (Figure 3D).

### 3.4. Gemcitabine enhances the antiproliferative effect of EBR in pancreatic cancer cell lines

**Figure 4:**
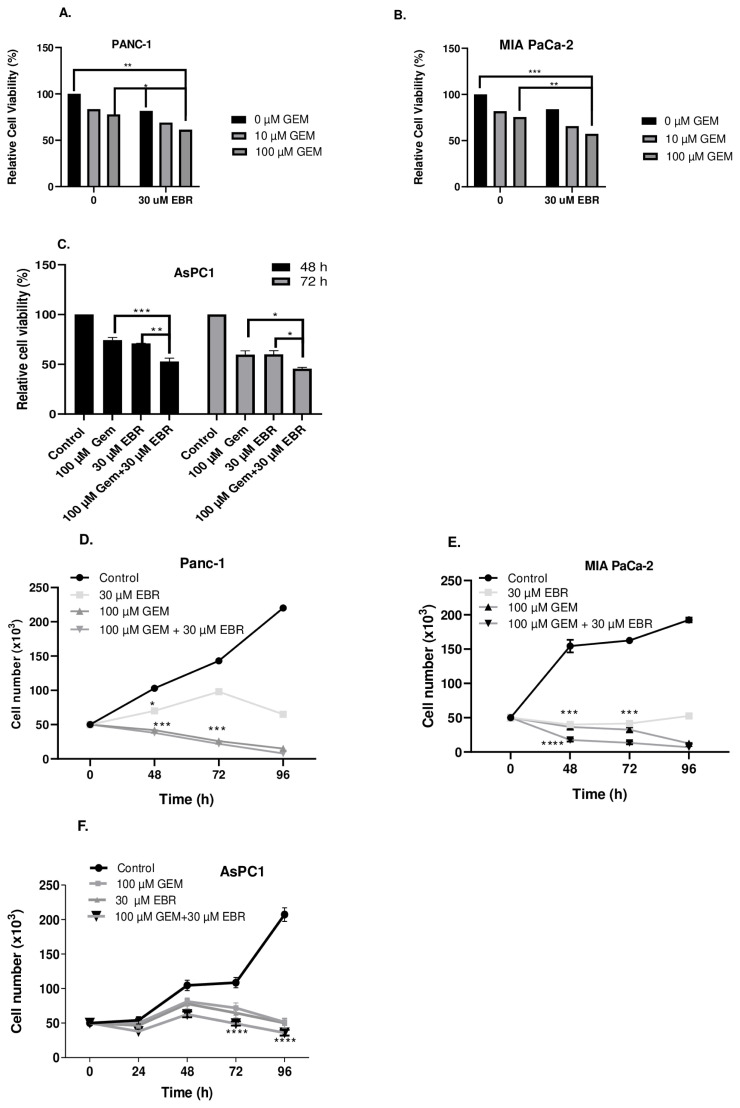

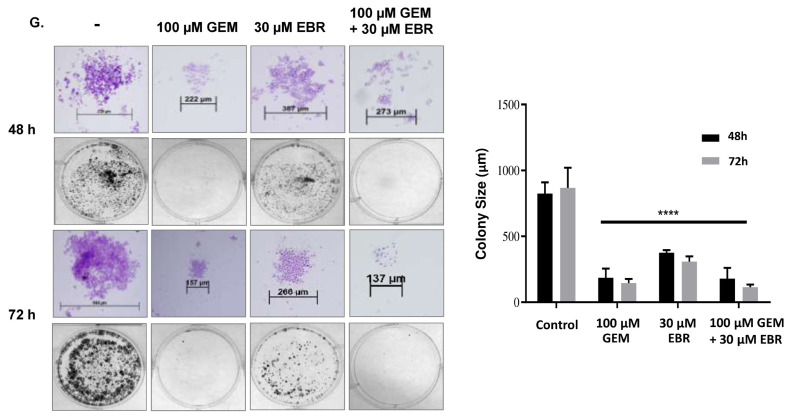
Cotreatment of EBR and gemcitabine decreased cell viability and inhibited cell proliferation of MIA PaCa-2, PANC-1, and AsPC-1 cells. A. PANC-1 and **B**. MIA PaCa-2 cells were treated with 10 and 100 μM gemcitabine with/without 30 μM EBR for 24 h. **C**. 100 μM gemcitabine and 30 μM EBR was applied both individually and in combination for 48 and 72 h in AsPC-1 cells. Subsequently, MTT cell viability assay was performed for each cell condition *p < 0.05, **p < 0.02, ***p < 0.001. **D**. PANC-1, **E**. MIA PaCa-2, and **F**. AsPC-1 cells were treated with indicated EBR and gemcitabine concentration both individually and in combination for 24, 48, 72, and 96 h. Next, a trypan blue assay was performed to investigate the effect of both gemcitabine and EBR on cell proliferation ***p < 0.001, ****p < 0.0001. **G**. Colony formation assay was performed in AsPC-1 cells following EBR and gemcitabine treatment for 48 and 72 h. Colony numbers were calculated for each condition ****p < 0.0001.

To investigate whether EBR could synergistically reduce cell proliferation with gemcitabine, a higher concentration of EBR (30 μM) was cotreated with both 10 and 100 μM gemcitabine for 24 h on MIA-PaCa-2, PANC-1, and AsPC-1 cells (Figures 4A–4C). Ten and 100 μM gemcitabine reduced cell viability almost by 15% and 20% in MIA PaCa-2 and PANC-1 cells. The ratios of cell viability of MIA PaCa-2 and PANC-1 cells were 75% and 60% following cotreatments of 30 μM EBR with 10 and 100 μM gemcitabine, respectively. As AsPC-1 cells are very aggressive, they were cotreated with 30 μM EBR and 100 μM gemcitabine for 48 and 72 h. It was observed that 100 μM gemcitabine treatment caused 30% and 40% cell viability loss for 48 and 72 h treatments, respectively. The combination of EBR and gemcitabine led to a further decrease of cell viability to 50% and 45%, respectively at 48 and 72 h treatments. The cell proliferation capacity of each pancreatic cancer cell was further inhibited by the cotreatment of EBR with gemcitabine (Figures 4D and 4F). The cytotoxic effect of 30 μM EBR and 100 μM gemcitabine was observed in each cell line in a time-dependent manner. The colony formation potential of AsPC-1 was determined following gemcitabine and EBR treatment at 48 and 72 h (Figure 4G). Gemcitabine had a remarkable effect on the inhibition of colony formation of AsPC-1 cells. While EBR treatment significantly reduced the colony numbers, cotreatment of EBR with gemcitabine further decreased the colony numbers and the diameter of colonies of AsPC-1 cells.

### 3.5. EBR augmented gemcitabine-induced apoptosis in a caspase-dependent manner in MIA PaCa-2 and PANC-1 cells

**Figure 5:**
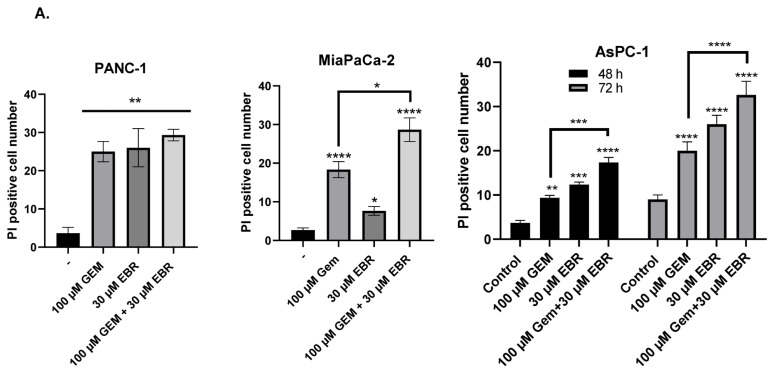

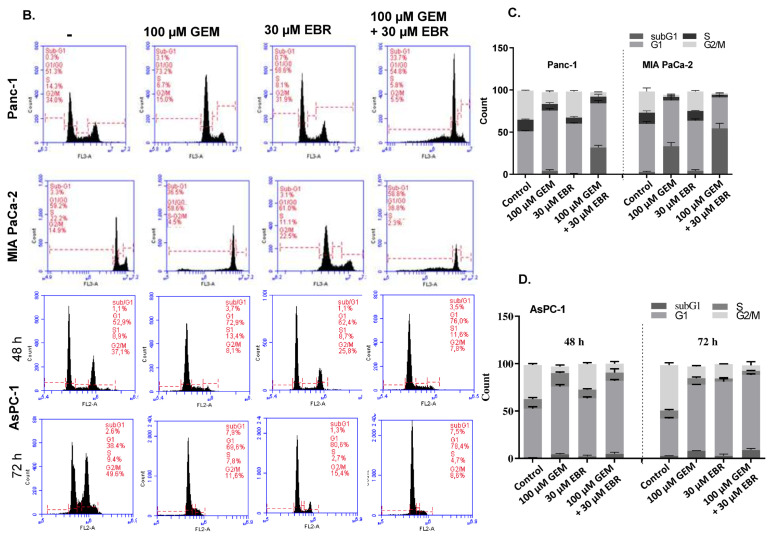

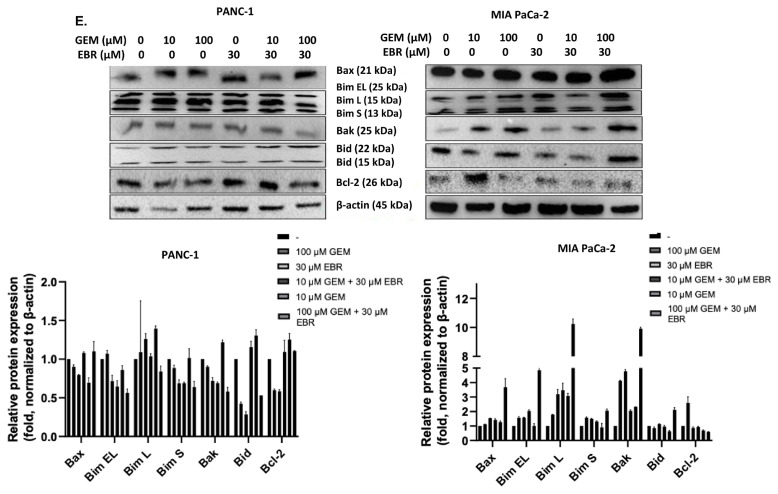

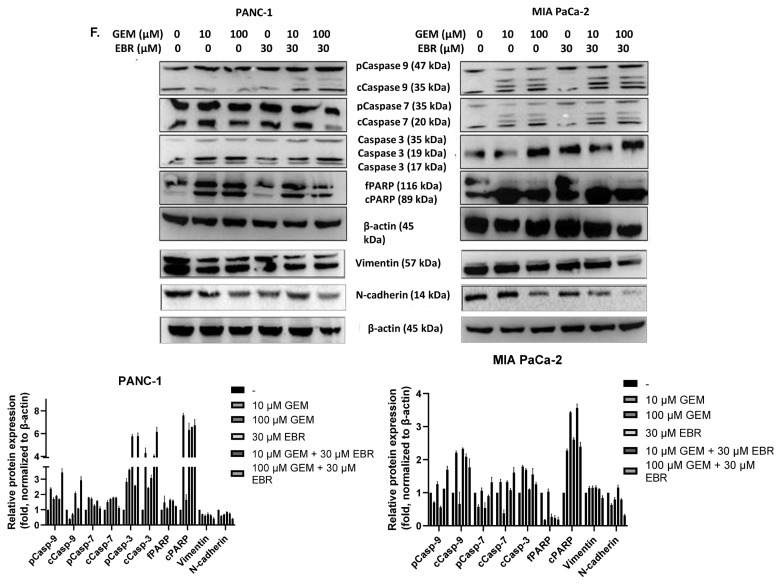

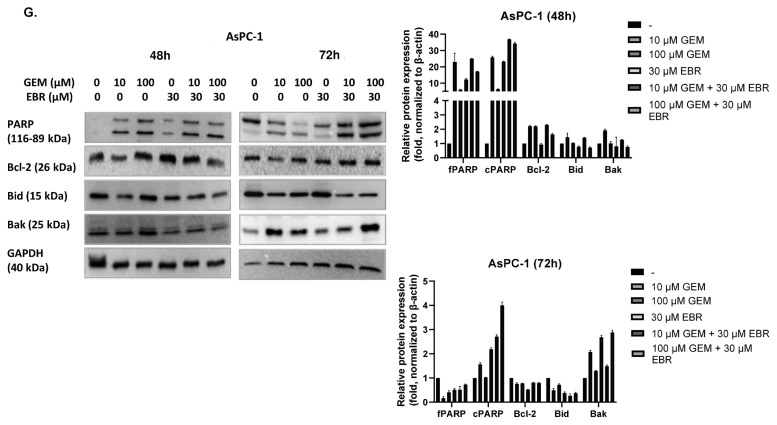
Cotreatment of EBR and gemcitabine had a potent effect on the induction of apoptosis in each pancreatic cancer cell. **A**. 100 μM gemcitabine and 30 μM EBR were applied both individually and in combination to PANC-1 (24 h), MIA PaCa-2 (24 h), and AsPC-1 cells (48 and 72 h). The cells were then stained by PI, and PI-positive cells were visualized by fluorescence microscopy and counted *p < 0.05, **p < 0.02, ***p < 0.001, ****p < 0.0001. **B**. At the same drug condition, flow cytometry analysis was performed following PI staining of PANC-1, MIA PaCa-2, and AsPC-1 cells. Proportions of each phase for **C**. PANC-1, MIA PaCa-2, **D**. and AsPC-1 cells are presented here. **E**. The cells were treated with 10 and 100 uM gemcitabine with/without 30 μM EBR. The expression levels of Bax, Bim, Bak, Bid, and Bcl-2; **F**. Caspase 9, caspase 7, caspase 3, Vimentin, and N-cadherin were analyzed by western blotting in PANC-1 and MIA PaCa-2 cells. β-actin was used as a loading control **G**. The expression levels of PARP, Bcl-2, Bid, and Bak were determined by western blotting following 10 and 100 μM gemcitabine with/without 30 μM EBR for 48 and 72 h in AsPC-1. GAPDH was used as a loading control.

PI staining was performed to observe the synergistic effect of cell death-inducing effect of the combination of EBR and gemcitabine treatment. The PI-stained (PI-positive cells) cells were counted (Figure 5A). Here, it was observed that cotreatment of EBR and gemcitabine resulted in a more significant increase in the cell death ratio when compared to alone treatment of gemcitabine and EBR in each pancreatic cancer cell line. Flow cytometric analysis following PI staining showed that gemcitabine treatment induced the cell cycle arrest at the G1 phase by 20% and there was no significant increase in the subG1 population in PANC-1 cells (Figure 5B). However, it was observed that the subG1 levels were 37% in MIA PaCa-2 cells treated with 100 μM gemcitabine. While 30 μM EBR treatment did not cause any effect on the cell cycle, the combination of EBR with gemcitabine increased significantly the apoptotic cell ratio to 33% and 58% in PANC-1 and MIA PaCa-2 cells, respectively (Figures 5B and 5C). The G2/M cell population seen at the 48-h and 72-h incubation of AsPC-1 cells was almost 37% and 50%, respectively. It was observed that the ratio of the G2/M population of AsPC-1 cells significantly decreased after 48 and 72 h treatments of gemcitabine. The combined treatment of EBR with gemcitabine increased the G1 cell cycle arrest in AsPC-1 cells (Figures 5B–5D). To further investigate the apoptotic potential of combined treatment, the Bax, Bim, Bak, Bid, and Bcl-2 levels were determined by immunoblotting (Figure 5E). The Bak, Bax, and cleaved Bid levels were increased and antiapoptotic Bcl-2 levels were decreased in a dose-dependent manner of gemcitabine treatment in PANC-1 cells. Although cleaved Bid and Bax levels were increased, it was observed that the Bak and Bim levels were decreased following 100 μM gemcitabine and 30 μM EBR treatments in PANC-1 cells. The cotreatment of 100 μM gemcitabine and 30 μM EBR led to a significant increase of proapoptotic Bax, Bim, Bak, and Bid expression levels in MIA PaCa-2 cells. Although EBR alone was determined to exert a modest apoptotic effect in the PANC-1 and MIA PaCa-2 cells at 30 μM concentration, when combined with 100 μM gemcitabine, it significantly enhanced apoptosis via cleavage of caspase-9, caspase-3, and PARP compared to untreated PANC-1 cells. The cotreatment of EBR with each concentration of gemcitabine increased the expression of cleaved caspase-9, caspase-7, and PARP in MIA PaCa-2 cells (Figure 5F). However, the full version of the caspase-3 level was higher in treated MIA PaCa-2 cells. The mesenchymal markers vimentin and N-cadherin were determined to understand the effect of gemcitabine and EBR combined treatment on the EMT potential of PANC-1 and MIA PaCa-2 cells. The higher expression level of cleaved PARP was correlated with the downregulation of both N-cadherin and vimentin expression levels following gemcitabine and EBR cotreatment in PANC-1 and MIA PaCa-2 cells. AsPC-1 cells were more resistant to EBR and gemcitabine treatment. Therefore, EBR and gemcitabine cotreatment was performed for 48 and 72 h. Afterward, the apoptotic effect was analyzed (Figure 5G). Gemcitabine-alone treatment increased the level of cleaved-PARP more than only EBR treatment in both 48 h and 72 h. Moreover, the combined treatment exerted higher cleaved-PARP levels for 48 h and 72 h in AsPC-1 cells. Bcl-2 levels were more significantly reduced by the combined treatment than both alone treatments of EBR and gemcitabine. The inactive form of Bid levels was decreased by the combined treatment, whereas there was no significant change in only EBR or gemcitabine treatment. While Bak levels were downregulated by cotreatment of EBR in 48 h, a significant increase was recorded in 72 h cotreatment of EBR and gemcitabine.

## 4. Discussion

Pancreatic cancer is one of the deadliest gastrointestinal malignancies. The prognosis of the disease is poor because of delayed detection and the limited effectiveness of therapies. Chemotherapy is the mainstream treatment against pancreas tumors, especially against unresectable ones. Although aggressive chemotherapy improves survival, nearly all patients develop drug resistance. Gemcitabine, an antimetabolite and deoxycytidine analog with antineoplastic activity, is used as first-line therapy alone or in combination. However, side effects such as myelosuppression and pulmonary toxicity, which occur in a very rapid way, restrict its usage. On the other hand, steroid administration is usually recommended to limit the side effects. EBR is a member of brassinosteroids (BR) with structural similarity to mammalian steroid hormones. Besides its critical roles in plant growth and antioxidant mechanisms, EBR is an apoptotic inducer in various cancer cells, but not in normal epithelial cells. EBR was first suggested as a nuclear hormone receptor (NHR) inhibitor; however, cells without NHR expression also undergo apoptotic cell death. Our studies indicated that ER stress induction was one of the main actions of EBR which was shown by stable isotope labeling by amino acids in cell culture (SILAC) and mass spectroscopy analyses (Obakan et al., 2015; Obakan-Yerlikaya et al., 2017). During ER stress, several signaling pathways are activated via ER membrane resident receptors PERK, IRE1α, and ATF6 to improve proper protein folding. If the stress cannot be handled after folding attempts, then the receptors can initiate apoptotic cell death. ER stress response regulation is an important phenomenon in terms of cancer cell death, survival, or chemoresistance. Therefore, in this study, we aimed to check the potential of EBR treatment to elevate gemcitabine response in pancreatic cancer cells concerning ER stress biomarkers. Our results indicated that EBR treatment had a significant effect on the reduction of cell viability of MIA PaCa-2, PANC-1, and AsPC-1 cells. As AsPC-1 cells had higher metastatic potential than MIA PaCa-2 and PANC-1, EBR treatment was carried out for 48 and 72 h. The cell viability reduction due to the EBR treatment was confirmed for the first time in the literature for pancreatic cancer cells with different genetic characteristics. This result was concluded as an effective approach for the induction of pancreatic cancer cell apoptosis since the heterogeneity of pancreatic cancer cells can lead to drug resistance and poor prognosis [15]. Therefore, in this study, the effect of EBR was investigated in a dose- and time-dependent manner in pancreatic cancer cells. Firstly, it was observed that 10 μM EBR inhibited the colony formation ability of both PANC-1 and MIA PaCa-2 cells, albeit 1 μM EBR treatment exerted different results on colony numbers of PANC-1 and MIA PaCa-2 cells. While 1 μM EBR caused a significant decrease in colony numbers of PANC-1. This result might be due to the different doubling times of the cell lines. According to a report by McIntyre and Kim, it is 28 h for PANC-1, whereas the original doubling time was reported as 52 h (Deer et al., 2010). Moreover, the cell proliferation rate of cells was differently affected by EBR treatment. Although the cell proliferation was inhibited by EBR treatment, after 48 h, the effect of EBR on the inhibition of cell proliferation was decreased in both MIA PaCa-2 and PANC-1 cells. The migration capacity of cells was prevented by EBR treatment. This result was found to be correlated with our previous ones which indicate that EBR can prevent wound healing and migration capacity of different cell lines (Obakan et al., 2014; Obakan et al., 2014; Coskun et al., 2015). The decrease in the wound healing and survival potential of cells indicated that they might be dead due to apoptotic induction. For this purpose and according to our previous studies indicating that EBR may induce apoptotic cell death mechanism in relation with ROS generation induction, we evaluated the induction of ROS in pancreatic cancer cells. We found that EBR led to an increase in ROS generation, which can be prevented by NAC cotreatment. Mitochondria are considered the main source of ROS and the increased rate of ROS generation in cells indicate defective mitochondria with unstable mitochondrial membrane potential, which usually occurs following chemotherapeutic agents exposure (Suski et al., 2012). The loss of mitochondrial membrane potential eventually causes cell death, mainly apoptosis (Zorov et al., 2006). The generation of ROS has been also associated with ER stress in various studies and it has been used as a therapeutic strategy in cancer models (Cao & Kaufman, 2014). It was recorded that EBR treatment induces ER stress mainly by targeting calreticulin in prostate cancer cells (Obakan-Yerlikaya et al., 2017). When pancreatic cancer was treated with EBR, ER stress is augmented in a dose-dependent manner and CALR and CALNX expressions were increased as suggested by our group in recent studies. EBR treatment-induced apoptotic cell death was found to be caspase-dependent in pancreatic cancer cells, supporting previous findings of EBR-induced cell death mechanism (Modi et al., 2016).

Although the low levels of EBR significantly reduced cell proliferation, we further investigated a higher dose of EBR in combination with gemcitabine because at this 30 μM concentration, EBR treatment caused a sharp cytotoxic effect in each pancreatic cancer cell line treated with gemcitabine and significantly reduced cell proliferation in a time-dependent manner. Gemcitabine concentrations were firstly selected according to the literature; however, further drug combination optimization clearly showed that 100 μM and 30 μM EBR concentrations were able to significantly reduce cell viability and cell proliferation. The cotreatment of EBR with gemcitabine had a remarkable effect on the inhibition of cell growth of PANC-1 and MIA PaCa-2 cells. AsPC-1 cells had a more drug-resistant profile than other pancreatic cancer cells (Awasthi et al., 2013). Therefore, the colony formation capacity of AsPC-1 cells was investigated following gemcitabine and EBR treatment. Although 100 μM gemcitabine decreased the colony numbers, EBR cotreatment led to a further decrease in colony formation capacity of AsPC-1 cells. However, the EBR and gemcitabine caused the increase of apoptotic cells in PANC-1 and MIA PaCa-2 cells, while there was no similar result in AsPC-1 cells. The combined treatment of new generation drugs with GEM yielded better results in terms of decrease in the clonogenic potential of pancreatic cancer cells (Di Matteo et al., 2019; Kwegyir-Afful et al., 2017). The increased apoptotic induction after cotreatment with EBR also affected the cell cycle phase distribution of pancreatic cancer cells. The G1 cell cycle arrest was observed following EBR and gemcitabine treatment in AsPC-1 cells. In addition, increased subG1 phase in all pancreatic cancer cells indicate further apoptotic induction. Gemcitabine, when combined with several anti-cancer agents showed the similar effect as EBR in pancreatic cells, including BH3 mimetic ABT-199, Chk1 inhibitors, or nabpaclitaxel (Bennett et al., 2012; Zhou et al., 2018; Passacantilli et al., 2018). The further apoptotic induction with EBR and gemcitabine cotreatment was also verified with the increase in the cleavages of caspase 9, caspase 3, and PARP levels in PANC-1 and MIA PaCa-2 cells. Moreover, the proapoptotic Bax and Bak levels were upregulated, whereas Bcl-2 as an antiapoptotic marker was downregulated in PANC-1 and MIA PaCa-2 cells. All these results verified that EBR is a mitochondria-mediated apoptotic agent that can elevate gemcitabine treatment in pancreatic cancer cells. Targeting mitochondria homeostasis has been suggested as an effective strategy to enhance chemosensitivity (Dubois et al., 2020). Therefore, it is not surprising that there are recent studies suggesting that the apoptotic effect of gemcitabine can be augmented by mitochondria-mediated apoptosis inducers including oblongifolin C, an autophagy flux inhibitor or doxycycline, a mitochondrial biogenesis inhibitor (Huang et al., 2020; Dijk et al., 2020). As investigated in previous studies, N-cadherin acts as a negative regulator of apoptosis and it can inhibit PARP cleavage to prevent apoptosis in various cancer cells (Nguyen et al., 2018). In addition, both downregulated N-cadherin and vimentin are important biomarkers of EMT process. While N-cadherin and vimentin are usually expressed at low level in epithelial cells, their aberrant expression is a very well-known phenomenon in breast, prostate, and pancreatic cancer cells, causing increased aggressiveness and metastasis (Fei et al., 2015; Mrozik et al., 2018). Our findings suggested that combined treatment of GEM and EBR decreased their expression profiles. As N-cadherin and vimentin endow cancer cells with increased migration and invasiveness, their further downregulation might suggest the inhibition of the metastatic potential of the cells.

Currently, the most commonly used treatments that involve combination partners with gemcitabine are 5-fluorouracil (5-FU): docetaxel, and cisplatin in pancreatic tumors (Heinemann, 2002). Combinations of gemcitabine with EBR have important potential to use in the treatment of pancreatic cancer. Although the combination of gemcitabine with EBR has demonstrated activity, and data showing a clear antisurvival benefit, we plan to check whether gemcitabine-induced chemoresistance can be achieved by EBR cotreatment and the role of ER stress during this process.

## References

[b1-turkjbiol-46-6-439] Awasthi N Zhang C Schwarz AM Hinz S Wang C 2013 Comparative benefits of nab-paclitaxel over gemcitabine or polysorbate-based docetaxel in experimental pancreatic cancer Carcinogenesis 34 10 2361 2369 10.1093/carcin/bgt227 23803690 PMC4023322

[b2-turkjbiol-46-6-439] Benham AM 2019 Endoplasmic Reticulum redox pathways: in sickness and in health The FEBS Journal 286 2 311 321 10.1111/febs.14618 30062765

[b3-turkjbiol-46-6-439] Bennett CN Tomlinson CC Michalowski AM Chu IM Luger D 2012 Cross-species genomic and functional analyses identify a combination therapy using a CHK1 inhibitor and a ribonucleotide reductase inhibitor to treat triple-negative breast cancer Breast Cancer Research 14 4 10.1186/BCR3230 PMC3680937 22812567

[b4-turkjbiol-46-6-439] Cao SS Kaufman RJ 2014 Endoplasmic reticulum stress and oxidative stress in cell fate decision and human disease Antioxidants and Redox Signaling Mary Ann Liebert Inc. Publisher 21 3 396 413 10.1089/ars.2014.5851 24702237 PMC4076992

[b5-turkjbiol-46-6-439] Coskun D Obakan P Arisan ED Çoker-Gürkan A Palavan-Ünsal N 2015 Epibrassinolide alters PI3K/MAPK signaling axis via activating Foxo3a-induced mitochondria-mediated apoptosis in colon cancer cells Experimental Cell Research 338 1 10 21 10.1016/J.YEXCR.2015.08.015 26318418

[b6-turkjbiol-46-6-439] De Wever O Demetter P Mareel M Bracke M 2008 Stromal myofibroblasts are drivers of invasive cancer growth International Journal of Cancer 123 10 2229 2238 10.1002/ijc.23925 18777559

[b7-turkjbiol-46-6-439] Deer EL González-Hernández J Coursen JD Shea JE Ngatia J 2010 Phenotype and Genotype of Pancreatic Cancer Cell Lines Pancreas 39 4 425 10.1097/MPA.0B013E3181C15963 20418756 PMC2860631

[b8-turkjbiol-46-6-439] Di Matteo S Nevi L Costantini D Overi D Carpino G 2019 The FXR agonist obeticholic acid inhibits the cancerogenic potential of human cholangiocarcinoma PloS One 14 1 10.1371/JOURNAL.PONE.0210077 PMC6345424 30677052

[b9-turkjbiol-46-6-439] Dijk SN Protasoni M Elpidorou M Kroon AM Taanman JW 2020 Mitochondria as target to inhibit proliferation and induce apoptosis of cancer cells: the effects of doxycycline and gemcitabine Scientific Reports 10 1 1 15 10.1038/s41598-020-61381-9 32152409 PMC7063048

[b10-turkjbiol-46-6-439] Dubois C Kondratskyi A Bidaux G Noyer L Vancauwenberghe E 2020 Co-targeting Mitochondrial Ca2+ Homeostasis and Autophagy Enhances Cancer Cells’ Chemosensitivity IScience 23 7 10.1016/J.ISCI.2020.101263 PMC7322071 32585596

[b11-turkjbiol-46-6-439] Fei F Zhang D Yang Z Wang S Wang X 2015 The number of polyploid giant cancer cells and epithelial-mesenchymal transition-related proteins are associated with invasion and metastasis in human breast cancer Journal of Experimental & Clinical Cancer Research: CR 34 1 10.1186/S13046-015-0277-8 PMC4690326 26702618

[b12-turkjbiol-46-6-439] Heinemann V 2002 Gemcitabine-based combination treatment of pancreatic cancer Seminars in Oncology 29 1 SUPPL 3 25 35 10.1053/sonc.2002.30749 11894005

[b13-turkjbiol-46-6-439] Huang Z Wang T Xia W Li Q Chen X 2020 Oblongifolin C reverses GEM resistance via suppressing autophagy flux in bladder cancer cells Experimental and Therapeutic Medicine 20 2 1431 10.3892/ETM.2020.8856 32765672 PMC7388549

[b14-turkjbiol-46-6-439] Kohli SK Bhardwaj A Bhardwaj V Sharma A Kalia N 2020 Therapeutic Potential of Brassinosteroids in Biomedical and Clinical Research Biomolecules 10 4 572 10.3390/BIOM10040572 PMC7226375 32283642

[b15-turkjbiol-46-6-439] Kwegyir-Afful AK Murigi FN Purushottamachar P Ramamurthy VP Martin MS 2017 Galeterone and its analogs inhibit Mnk-eIF4E axis, synergize with gemcitabine, impede pancreatic cancer cell migration, invasion and proliferation and inhibit tumor growth in mice Oncotarget 8 32 52381 52402 10.18632/ONCOTARGET.14154 28881737 PMC5581036

[b16-turkjbiol-46-6-439] Lin Y Jiang M Chen W Zhao T Wei Y 2019 Cancer and ER stress: Mutual crosstalk between autophagy, oxidative stress and inflammatory response Biomedicine and Pharmacotherapy Elsevier Masson SAS 118 109249 10.1016/j.biopha.2019.109249 31351428

[b17-turkjbiol-46-6-439] McGuigan A Kelly P Turkington RC Jones C Coleman HG 2018 Pancreatic cancer: A review of clinical diagnosis, epidemiology, treatment and outcomes World Journal of Gastroenterology Baishideng Publishing Group Co., Limited 24 43 4846 4861 10.3748/wjg.v24.i43.4846 30487695 PMC6250924

[b18-turkjbiol-46-6-439] Melisi D Garcia-Carbonero R Macarulla T Pezet D Deplanque G 2018 Galunisertib plus gemcitabine vs. gemcitabine for first-line treatment of patients with unresectable pancreatic cancer British Journal of Cancer 119 10 1208 1214 10.1038/s41416-018-0246-z 30318515 PMC6251034

[b19-turkjbiol-46-6-439] Modi S Kir D Banerjee S Saluja A 2016 Control of Apoptosis in Treatment and Biology of Pancreatic Cancer Journal of Cellular Biochemistry 117 2 279 288 10.1002/jcb.25284 26206252 PMC5724757

[b20-turkjbiol-46-6-439] Mrozik KM Blaschuk OW Cheong CM Zannettino ACW Vandyke K 2018 N-cadherin in cancer metastasis, its emerging role in haematological malignancies and potential as a therapeutic target in cancer BMC Cancer 18 1 1 16 10.1186/S12885-018-4845-0 30285678 PMC6167798

[b21-turkjbiol-46-6-439] Nguyen PT Nguyen D Chea C Miyauchi M Fujii M 2018 Interaction between N-cadherin and decoy receptor-2 regulates apoptosis in head and neck cancer Oncotarget 9 59 31516 31530 10.18632/oncotarget.25846 30140387 PMC6101147

[b22-turkjbiol-46-6-439] Nolan TM Vukasinović N Liu D Russinova E Yin Y 2020 Brassinosteroids: Multidimensional regulators of plant growth, development, and stress responses Plant Cell 32 2 298 318 10.1105/tpc.19.00335 PMC7008487 31776234

[b23-turkjbiol-46-6-439] Obakan-Yerlikaya P Arisan ED Coker-Gurkan A Adacan K Ozbey U 2017 Calreticulin is a fine tuning molecule in epibrassinolide-induced apoptosis through activating endoplasmic reticulum stress in colon cancer cells Molecular Carcinogenesis 56 6 1603 1619 10.1002/MC.22616 28112451

[b24-turkjbiol-46-6-439] Obakan P Arisan ED Calcabrini A Agostinelli E Bolkent Ş 2014 Activation of polyamine catabolic enzymes involved in diverse responses against epibrassinolide-induced apoptosis in LNCaP and DU145 prostate cancer cell lines Amino Acids 46 3 553 564 10.1007/S00726-013-1574-1 23963538

[b25-turkjbiol-46-6-439] Obakan P Arisan ED Coker-Gurkan A Palavan-Unsal N 2014 Epibrassinolide-induced apoptosis regardless of p53 expression via activating polyamine catabolic machinery, a common target for androgen sensitive and insensitive prostate cancer cells The Prostate 74 16 1622 1633 10.1002/PROS.22879 25214240

[b26-turkjbiol-46-6-439] Obakan P Barrero C Coker-Gurkan A Arisan ED Merali S 2015 SILAC-Based Mass Spectrometry Analysis Reveals That Epibrassinolide Induces Apoptosis via Activating Endoplasmic Reticulum Stress in Prostate Cancer Cells PloS One 10 9 10.1371/JOURNAL.PONE.0135788 PMC4564160 26353013

[b27-turkjbiol-46-6-439] Oettle H Riess H 2002 Gemcitabine in combination with 5-fluorouracil with or without folinic acid in the treatment of pancreatic cancer Cancer 95 4 SUPPL 912 922 10.1002/cncr.10758 12209671

[b28-turkjbiol-46-6-439] Passacantilli I Panzeri V Terracciano F Fave GD Sette C 2018 Co-Treatment with gemcitabine and nab-paclitaxel exerts additive effects on pancreatic cancer cell death Oncology Reports 39 4 1984 1990 10.3892/OR.2018.6233/HTML 29393478

[b29-turkjbiol-46-6-439] Pelosi E Castelli G Testa U 2017 Pancreatic cancer: Molecular characterization, clonal evolution and cancer stem cells Biomedicines MDPI AG 5 4 10.3390/biomedicines5040065 PMC5744089 29156578

[b30-turkjbiol-46-6-439] Plunkett W Huang P Xu YZ Heinemann V Grunewald R 1995 Gemcitabine: Metabolism, mechanisms of action, and self-potentiation Seminars in Oncology 22 4 SUPPL 11 3 10 7481842

[b31-turkjbiol-46-6-439] Sommariva M Gagliano N 2020 E-Cadherin in Pancreatic Ductal Adenocarcinoma: A Multifaceted Actor during EMT Cells 9 4 1040 10.3390/cells9041040 32331358 PMC7226001

[b32-turkjbiol-46-6-439] Suski JM Lebiedzinska M Bonora M Pinton P Duszynski J 2012 Relation between mitochondrial membrane potential and ROS formation Mitochondrial Bioenergetics Methods and Protocols 810 183 205 10.1007/978-1-61779-382-0_12 22057568

[b33-turkjbiol-46-6-439] Wang S Kaufman RJ 2012 The impact of the unfolded protein response on human disease Journal of Cell Biology The Rockefeller University Press 197 7 857 867 10.1083/jcb.201110131 22733998 PMC3384412

[b34-turkjbiol-46-6-439] Zhou Y Liu H Xue R Tang W Zhang S 2018 BH3 Mimetic ABT-199 Enhances the Sensitivity of Gemcitabine in Pancreatic Cancer in vitro and in vivo Digestive Diseases and Sciences 63 12 3367 3375 10.1007/S10620-018-5253-7 30155839

[b35-turkjbiol-46-6-439] Zorov DB Juhaszova M Sollott SJ 2006 Mitochondrial ROS-induced ROS release: An update and review Biochimica et Biophysica Acta (BBA) - Bioenergetics 1757 5–6 509 517 10.1016/J.BBABIO.2006.04.029 16829228

